# Effects of Concentrated Long-Chain Omega-3 Polyunsaturated Fatty Acid Supplementation on Quality of Life after Radical Prostatectomy: A Phase II Randomized Placebo-Controlled Trial (RCT-EPA)

**DOI:** 10.3390/nu15061369

**Published:** 2023-03-11

**Authors:** Hanane Moussa, Karine Robitaille, Jean-François Pelletier, Roxane Tourigny, Yves Fradet, Louis Lacombe, Paul Toren, Michele Lodde, Rabi Tiguert, Thierry Dujardin, Yves Caumartin, Thierry Duchesne, Pierre Julien, Josée Savard, Caroline Diorio, Vincent Fradet

**Affiliations:** 1 CHU de Québec-Université Laval Research Center, Québec, QC G1R 3S1, Canada; 2 Institute of Nutrition and Functional Foods (INAF) and NUTRISS Center—Nutrition, Health and Society of Université Laval, Québec, QC G1V 0A6, Canada; 3 Centre de Recherche sur le Cancer de l’Université Laval, Québec, QC G1R 3S3, Canada; 4 Centre Intégré de Cancérologie du CHU de Québec-Université Laval, Québec, QC G1J 5B3, Canada; 5 Department of Mathematics and Statistics, Université Laval, Québec, QC G1V 0A6, Canada; 6 School of Psychology, Université Laval, Québec, QC G1V 0A6, Canada

**Keywords:** fish oil, eicosapentaenoic acid (EPA), prostate cancer, prostate-specific quality of life, surgery

## Abstract

Prostate cancer (PCa) and associated treatments incur symptoms that may impact patients’ quality of life. Studies have shown beneficial relationships between diet, especially omega-3 fatty acids, and these symptoms. Unfortunately, only few data describing the relationship between long-chain omega-3 fatty acids (LCn3) and PCa-related symptoms in patients are available. The purpose of this study was to evaluate the effects of LCn3 supplementation on PCa-specific quality of life in 130 men treated by radical prostatectomy. Men were randomized to receive a daily dose of either 3.75 g of fish oil or a placebo starting 7 weeks before surgery and for up to one-year post-surgery. Quality of life was assessed using the validated EPIC-26 and IPSS questionnaires at randomization, at surgery, and every 3 months following surgery. Between-group differences were assessed using linear mixed models. Intention-to-treat analyses showed no significant difference between the two groups. However, at 12-month follow-up, per-protocol analyses showed a significantly greater increase in the urinary irritation function score (better urinary function) (MD = 5.5, *p* = 0.03) for the LCn3 group compared to placebo. These results suggest that LCn3 supplementation may improve the urinary irritation function in men with PCa treated by radical prostatectomy and support to conduct of larger-scale studies.

## 1. Introduction

Prostate cancer (PCa) is the most common cancer among men in Canada, with 24,600 men diagnosed in 2022, representing 20% of all new cancer cases [1,2]. PCa and related treatments are associated with many symptoms affecting patients’ quality of life, including erectile dysfunction, urinary problems, pain, fatigue, and psychological distress. PCa costs to Canadian healthcare system more than $3.89 billion annually [3], illustrating the important individual and social burden of this disease [1].

Radical prostatectomy is one of the main treatments for men diagnosed with intermediate- to high-grade PCa [4]. Unfortunately, this treatment is associated with several adverse effects affecting patients’ quality of life, such as sexual dysfunction and urinary incontinence [5]. Given the excellent survival rates from PCa [6], the quality of life of these patients is a major concern during and after cancer treatment. For these patients, nutritional interventions are considered accessible and low-cost strategies to improve health and quality of life [7]. Long-chain omega-3 fatty acid (LCn3) supplementation represents a promising strategy to improve the quality of life of patients with PCa based on its anti-inflammatory properties. Indeed, LCn3 supplementation in advanced cancer patients may reduce inflammation by suppressing the synthesis of targeted cytokines [8,9,10,11], which are likely involved in several quality-of-life functions [12,13,14,15,16]. Previous epidemiological studies showed that serum C-reactive protein level, a marker of inflammation induced by cytokines, is associated with increased storage, lower urinary tract symptoms, and urgency incontinence [16,17].

The impact of LCn3 on PCa-specific quality of life has sparsely been studied. Only a few studies evaluated the relationships between LCn3-related dietary habits and prostate-specific symptoms. One study assessing PCa-specific quality of life showed that higher fish consumption, the main source of LCn3, after diagnosis was associated with higher urinary irritation/obstruction scores (better urinary function), while no association with urinary incontinence and sexual function was found [18]. In men with benign prostatic hypertrophy, the frequency of fish, meat, and egg consumption was inversely correlated with lower urinary tract symptoms [19]. Other studies suggested that patients without erectile dysfunction had a dietary pattern richer in fish, fruits, vegetables, nuts, and whole grains but lower in red and processed meat than men with erectile dysfunction [20]. On the other hand, one study found no association between eicosapentaenoic acid (EPA) intake, a subtype of LCn3, and lower urinary tract symptoms [21]. Importantly, we found no prospective trial having evaluated the causal effect of LCn3 supplementation on PCa-specific quality of life in a population of men treated for a PCa. Our objective was to determine the effects of a daily LCn3 supplementation on PCa-specific quality of life in patients treated by radical prostatectomy. This is a planned secondary endpoint of the phase II randomized placebo-controlled trial.

## 2. Materials and Methods

### 2.1. Study Population and Design

The study is a randomized, placebo-controlled, double-blind trial in men treated by radical prostatectomy for an aggressive localized PCa (Gleason score ≥ 7, International Society of Urological Pathology (ISUP) Grade group ≥ 2 [22]). To be eligible, participants had to be 18 years of age or older, have chosen a radical prostatectomy for the primary PCa treatment, and have no intolerance or allergy to fish or sunflower. Patients already taking omega-3 supplements could participate after a washout period of at least eight weeks before randomization. Other supplements had to be discontinued for the entire intervention period.

The study protocol has been published previously [23]. Briefly, participants were randomized to either 3.75 g per day of fish oil rich in EPA (MAG-EPA) or 3.75 g of sunflower oil rich in oleic acid (placebo), started on average 7 weeks (4–10 weeks) before radical prostatectomy. The supplementation was pursued for one year after radical prostatectomy. The randomization was generated using permuted random blocks of size 2–8 before the start of the study and kept concealed for all study personnel and patients for the duration of the study. The randomization was managed by the CHU de Québec-Université Laval clinical research oncology pharmacy after confirmation of eligibility. The pharmacy personnel counted the adherence to intervention [24] by counting the remaining capsules reported by the participants every 3 months. Participants were also seen by a research nurse every 3 months for follow-up. Adverse events assessment was completed for each visit. Quality of life was measured using validated questionnaires, as described below.

### 2.2. Intervention

Participants were randomized into two groups (ratio 1:1). Those assigned to the MAG-EPA intervention group (n = 65) received a daily dose of six capsules of 625 mg each of fish oil (as monoglyceride fatty acids containing 90% omega-3, of which 75% EPA and 10% DHA) for a total of 3.3 g LCn3 daily. Participants assigned to the placebo group (n = 65) received a daily dose of placebo in six capsules identical in appearance and taste, containing sunflower oil rich in oleic acid (HOSO). These capsules contained only traces of omega-6 fatty acids (no trace of omega-3) and approximately 82% omega-9 fatty acids (oleic acid). HOSO is a biologically neutral oil on inflammation and has therefore been used as a placebo in at least three clinical trials on the effects of LCn3 [25,26,27].

### 2.3. Fatty Acid Profiles

Fatty acid profiles in red blood cell membranes were determined by gas chromatography–mass spectrometry as described previously [28] at study baseline and 12 months after surgery. Fatty acids are expressed as relative percentages of total fatty acids.

### 2.4. Prostate-Specific Quality of Life

Aspects of prostate-specific quality of life were assessed at randomization and every 3 months up to one year after surgery, using the International Prostate Symptom Score (IPSS) and the Expanded Prostate Cancer Index Composite (EPIC-26) questionnaires for which the French-Canadian versions have been validated [29,30,31]. Questions in these two questionnaires were evaluated the previous month. The IPSS contains seven multiple-choice questions on voiding and storage difficulties for which the severity is rated on a scale of 0–5 points for a total of 35 points maximum. A low score means fewer voiding difficulties. The EPIC-26 contains 26 multiple-choice questions measuring the following five specific quality-of-life domains: urinary incontinence, urinary irritation, bowel function, hormonal function, and sexual function. Each domain is scored on a scale of 0–100. Higher scores indicate better function. The psychometric properties of these questionnaires have been well demonstrated [31,32].

### 2.5. Potential Confounders

Potential confounders were measured at study baseline before the intervention began. Potential confounders were identified from the literature and included age, education, income, smoking, physical activity, ethnicity, comorbidity, living alone, body mass index (BMI), PCa stage, PCa grade, and prostate-specific antigen (PSA). The participants‘ diet was also measured using a validated online food frequency questionnaire (Web-FFQ) [33].

### 2.6. Statistical Analysis

We applied a longitudinal analysis to investigate repeated outcome measures after treatment for PCa. Linear mixed models were used to assess between-group differences in changes in quality of life. Differences between the mean quality of life scores (DM) of the two treatment groups and their 95% confidence intervals were measured. To account for within-subject dependence, residual correlations between observations were modeled using an unstructured type matrix. Sensitivity analyses were also performed using compound symmetry (type CS) and autoregressive type 1 AR matrices (1). Based on the Akaike information criterion (AIC), the unstructured type matrix was the best fit for the data. We used an intention-to-treat approach, including all patients for whom the targeted quality of life score could be calculated. Per-protocol analyses were also performed among patients who adhered to the intervention by taking at least 80% of their capsules at each visit. Because of the unbalanced distribution, analyses were adjusted for BMI and for the National Comprehensive Cancer Network (NCCN) risk, which considers the PSA level, cancer grade, and cancer stage, all together. Age distribution was not perfectly balanced between the two groups (*p* = 0.13), and its addition to the model changed the beta coefficient by more than 20%. It was then included in the models. To mitigate bias related to missing data due to attrition, inverse probability of censoring weights was performed. We also performed sensitivity analyses by controlling for EPA level at randomization and by excluding men taking medication for urinary symptoms or erectile dysfunction during the study. Statistical testing was two-sided, with the threshold of statistical significance at *p* < 0.05. All statistical analyses were performed using SAS version 9.4.

## 3. Results

**Figure 1:**
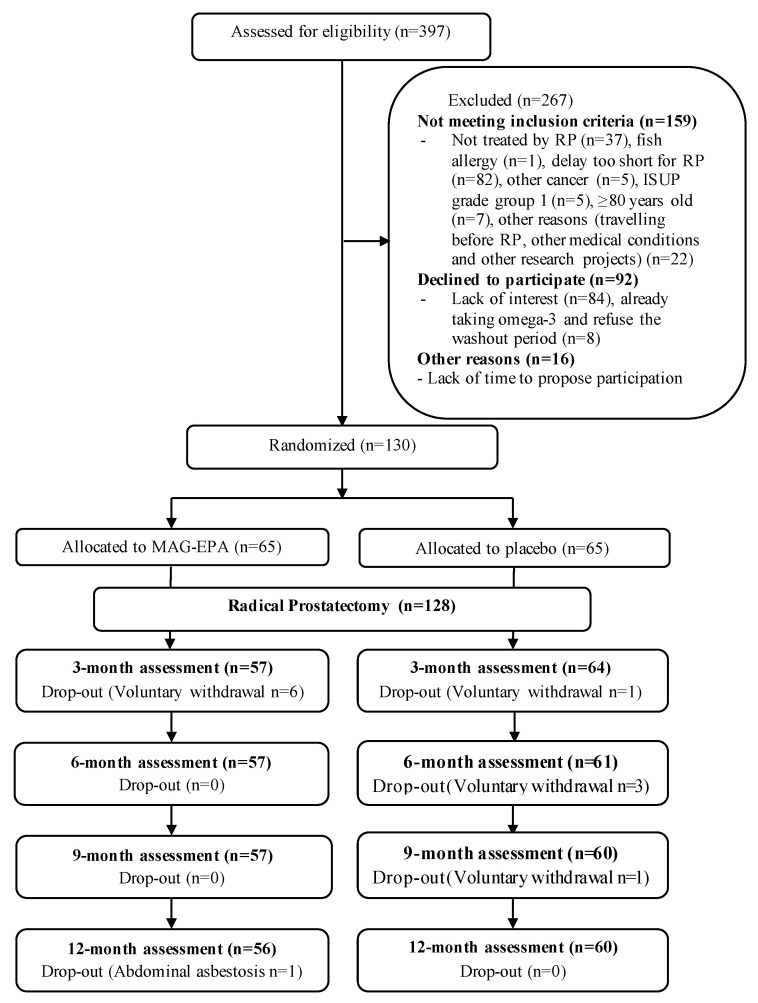
Study’s flow chart. MAG-EPA: monoacylglyceride-conjugated eicosapentaenoic acid; RP: radical prostatectomy; ISUP: International Society of Urological Pathology.

Of the 397 PCa patients assessed for eligibility (Figure 1), 130 patients were enrolled between February 2015 and June 2017.

There were no major differences in patients’ baseline characteristics between the two study groups, except for BMI, grade group, and NCCN risk. Indeed, 35% of participants had a BMI greater than 30, and 11% had a BMI less than 25 in the MAG-EPA group, compared to 23% and 25%, respectively, in the placebo group. In addition, 31% of participants in the MAG-EPA group were at high risk, according to the NCCN, compared to 15% in the placebo group (Table 1).

At randomization, participants from both groups had high quality-of-life scores for all quality-of-life domains except for sexual function. The following mean scores were comparable between groups, except for urinary irritation function and bowel function: the mean score of urinary irritation function was 83.97 for the MAG-EPA group versus 87.50 for the placebo group (*p* = 0.17), while bowel function had a mean score of 90.71 for the MAG-EPA group versus 93.88 for placebo (*p* = 0.08) (Table 1).

Associations between quality-of-life domains and the intervention using an intention-to-treat approach are presented in Table 2, specifically the mean score for both groups, including the mean difference between and within the two groups at each visit. As expected, at a 3-month post-radical prostatectomy, the mean score for urinary incontinence, urinary irritation, and sexual function deteriorated in both groups, with a more pronounced deterioration in the MAG-EPA group.

At the 12-month follow-up, the urinary irritation score was significantly improved by 5.6 (*p* = 0.007) for the MAG-EPA group compared to randomization, versus an increase of 1.0 (*p* = 0.62) for the placebo group, with a mean difference between groups of 3.5 (*p* = 0.11) (Table 2). The same result was obtained with the per-protocol analysis, showing a significant mean difference between groups of 5.5 (*p* = 0.03) (Table 3). These associations remained similar following the sensitivity analyses after adjustment for EPA level at randomization. However, those were slightly attenuated after the exclusion of men who took medications during the study to treat urinary symptoms (DM = 5.2; *p* = 0.06) (data not shown).

Finally, bowel function was significantly improved at 12 months compared to randomization for the MAG-EPA group (intention-to-treat analysis DM = 3.8, *p* = 0.009). However, no difference was observed between groups (DM = −0.6, *p* = 0.69) (Table 2). A similar result was obtained with the per-protocol analysis (Table 3).

Reported adverse events for the entire study in patients who underwent radical prostatectomy are presented in Table 4. Reported adverse events were expected, and their frequencies were <5% for all reported symptoms and similar between groups. The most common were diarrhea, skin rash, and nausea (n = 4 for each adverse event). During the entire study, 7 patients (10.8%) in the placebo group and 10 (15.9%) in the MAG-EPA group had at least one adverse event. Withdrawal from the study because of adverse events occurred in 1 patient (1.5%) in the placebo compared to 5 (7.9%) in the MAG-EPA group.

Adherence to the intervention was excellent. We measured an adherence between 83 and 86% when considering a most severe definition (assuming a zero value for missing data) and 93% when using exact data available (Table 5).

**Figure 2:**
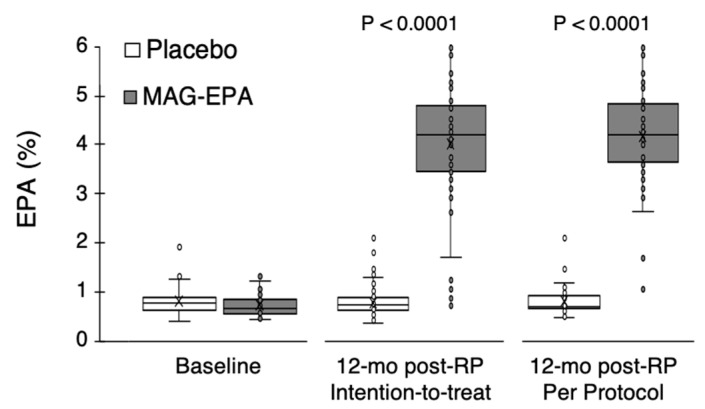
EPA level in red blood cell membranes of patients. EPA level is expressed as a relative percentage of total fatty acids, at the study baseline (n = 130) and after one year following RP, for the intention to treat population (n = 116) and the per-protocol population (n = 87 patients who took at least 80% of capsules). *p*-values were obtained using the Wilconxon test. RP: radical prostatectomy. MAG-EPA: monoacylglyceride-conjugated eicosapentaenoic acid.

The EPA level in red blood cell membranes of the MAG-EPA group was significantly increased at 12 months (4%), while it was stable in the placebo group (0.8%) (Figure 2).

## 4. Discussion

This is the first double-blind, placebo-controlled clinical trial investigating the effect of LCn3 supplementation, mainly EPA, on PCa-specific quality of life, which is commonly affected in men undergoing prostatectomy. Our results indicated a significant increase in the urinary irritation score (better urinary function, EPIC-26) between randomization and the 12-month follow-up in the MAG-EPA group, particularly in the per-protocol analysis. A significant improvement in voiding and storage function (decrease in IPSS score) was observed at 12 months of follow-up for both groups, but the greater improvement in the MAG-EPA group was not statistically significant.

Only a few studies evaluated the effect of LCn3 on quality of life. One observational study evaluated the effect of the Mediterranean diet, including fish, on the quality of life of PCa patients, from whom 54% were treated by radical prostatectomy [18]. This prospective study showed that a higher fish consumption after diagnosis was associated with slightly fewer urinary irritation symptoms (*p*-trend = 0.05). This is consistent with our results, showing that the MAG-EPA group had higher urinary irritation scores by an average of 5.5 points compared to the placebo at the 12-month follow-up. According to the literature, this 5.5 difference represents a clinically significant change (between 5 and 7 points) in the urinary irritation function between MAG-EPA and placebo [34]. The mechanism by which a supplementation of LCn3 improves urinary irritation in men treated by radical prostatectomy is still unclear but could be related to the attenuation of inflammation, which is considered to be one cause of urinary symptoms.

In our study, LCn3 was not associated with significantly greater improvements in voiding symptoms as measured by the IPSS compared to the placebo. This finding is consistent with the only observational study that found no association between EPA and lower urinary tract symptoms [21]. The improvement in voiding symptoms was observable in both groups over time, as expected during post-operative recovery. The lack of LCn3 (MAG-EPA) effect on voiding symptoms could also be related to participants who had no or only minor voiding problems at randomization in both groups (floor effect). The improvement observed in the IPSS score in the placebo group may be related to the placebo effect observed in previous studies. Indeed, a randomized controlled trial in patients with enlarged prostate evaluated the effect of tamsulosin, treatment of benign hypertrophy, compared to the placebo found that the mean IPSS score was decreased by 5.78 for the placebo group after 12 months follow-up [35]. This may also explain the lack of significant differences between the two groups in our study.

LCn3 supplementation was not associated with neither urinary incontinence nor sexual function improvements compared to placebo. These results are surprising, given prior evidence suggesting that lifestyle habits targeting inflammation may have a role in reducing the burden of sexual dysfunction and urinary incontinence [12,13,14,15,16]. However, our results are consistent with a previous study that found no association between fish consumption and these two symptom domains [18], which are mostly affected by the radical prostatectomy. A previous study highlighted that urinary incontinence and sexual dysfunction were the two most common unresolved drawbacks up to one year after surgery, suggesting that a longer recovery follow-up is needed [36]. Late functional recovery is sometimes observed in the clinic. These events were not captured given the 12-month duration of our study. Finally, we observed a slightly greater deterioration in urinary incontinence and sexual function scores in the MAG-EPA group. These differences were not statistically significant. Since more high-risk PCa cases were randomized in the MAG-EPA group, it is possible that longer and more invasive surgeries may have had a more functional impact. Adjustment for PCa risk and BMI, another factor affecting the complexity of prostatectomy, was performed, but residual confounding, which would hamper our ability to identify a MAG-EPA effect, cannot be excluded.

This study had several strengths worth mentioning. The methodology included a randomized, double-blind trial design. This effort for blinding provides confidence that the observed differences are not biased. This trial was also placebo-controlled. The greater placebo effect, being well documented on such functional outcomes and observed herein on the urinary irritation domain, is a notable clinically significant finding. The biological plausibility was supported by a very significant increase in the EPA level in red blood cell membranes of the MAG-EPA group over the course of the study, while it was stable in the placebo group. This also represents a biological measure of compliance [24], supporting that the intervention was globally well tolerated. Loss to follow-up was minimal and similar in both groups (13.9% vs. 7.7% over 12 months in the MAG-EPA and placebo, respectively, Figure 1), providing confidence that attrition bias is unlikely. To reduce the possibility of desirability bias, all questionnaires were anonymous, and participants were informed that research professionals highly valued the protection of data privacy.

On the other hand, some limitations are worth mentioning. The relatively small sample size also limited our statistical power and may explain some of the null results found. However, other small trials have found significant findings on urinary function, such as one of 63 patients [37]. Finally, our study population generally had a relatively healthy basic diet. For example, the mean omega-6:omega-3 dietary fatty acid ratio at baseline was 6.3 ± 1.8, far from that of a typical western diet up to 15–20 [38,39]. Our trial did not use dietary intake parameters as selection criteria. It is possible that selecting patients with lower LCn3 dietary intake would have provided a greater opportunity for the impact of MAG-EPA on LCn3 and on functional outcomes. There were 23% missing data for dietary intake at randomization, precluding its use as an adjustment factor. However, the comparison of the available data showed no significant difference in LCn3 dietary intake between the two groups.

## 5. Conclusions

The intention-to-treat analysis of this phase II placebo-controlled trial did not support a beneficial effect of LCn3 supplementation on the overall quality of life of PCa patients. However, the per-protocol analysis suggests that a daily supplementation with 3.3 g LCn3 for one year improves urinary irritation function in men with PCa treated by radical prostatectomy. As this is the first randomized trial examining the impact of LCn3 on prostate-specific quality of life in men with PCa, larger trials are warranted to examine the replicability of our findings.

## Figures and Tables

**Table 1:** Baseline characteristics of participants.

Variables	MAG-EPA (n = 65)	Placebo (n = 65)	*p*-Value
**Socio-demographic characteristics**			
Age (years)			0.13 ^1^
Mean (SD)	64.34 (6.31)	62.50 (7.36)	
Median (Q1–Q3)	65.0 (60.0–70.0)	64.0 (58.0–67.0)	
BMI (Kg/m^2^), n (%)			0.06 ^3^
<25	7 (10.77)	16 (24.62)	
25–29	34 (52.31)	32 (49.23)	
>= 30	23 (35.38)	15 (23.08)	
Missing	1 (1.54)	2 (3.08)	
Smoking status n (%)			0.30 ^3^
Current	4 (6.15)	8 (12.31)	
Former	34 (52.31)	27 (41.54)	
Never	26 (40.00)	30 (46.15)	
Missing	1 (1.54)	0 (0)	
Education, n (%)			0.42 ^3^
Secondary school or less	24 (36.92)	19 (29.23)	
Postsecondary diploma	18 (27.69)	25 (38.46)	
University degree	21 (32.31)	20 (30.77)	
Missing	2 (3.08)	1 (1.54)	
Physical activity n (%)			0.74 ^3^
Active	24 (36.92)	27 (41.54)	
Inactive	37 (56.92)	37 (56.92)	
Missing	4 (6.15)	1 (1.54)	
Marital status n (%)			0.84 ^3^
Married or common-law	54 (83.08)	54 (83.08)	
Single or not married	10 (15.38)	11 (16.92)	
Missing	1 (1.54)	0 (0)	
**Medical characteristics**			
PSA (ng/mL)			0.19 ^2^
Mean (SD)	8.74 (9.33)	6.64 (5.53)	
Median (Q1–Q3)	6.00 (4.40–8.70)	5.70 (4.00–7.00)	
Grade group n (%)			0.03 ^3^
2 (3 + 4)	31 (47.69)	41 (63.08)	
3 (4 + 3)	17 (26.15)	18 (27.69)	
>=4 (8 and 9)	17 (26.15)	6 (9.23)	
Cancer Stage n (%)			0.17 ^3^
T2a or less	52 (80.00)	59 (90.77)	
T2b or T2c	4 (6.15)	3 (4.62)	
T3 or more	9 (13.85)	3 (4.62)	
NCCN risk, n (%)			0.04 ^3^
Intermediate risk (2)	45 (69.23)	55 (84.62)	
High risk (3)	20 (30.77)	10 (15.38)	
Comorbidity index n (%)			0.62 ^3^
0	41 (63.08)	39 (60.00)	
1	10 (15.38)	15 (23.08)	
≥2	10 (15.38)	11 (16.92)	
Missing	4 (6.15)	0 (0)	
RBC fatty acid profile (%) *			
Total n3			0.98 ^1^
Mean (SD)	7.40 (1.17)	7.40 (1.02)	
Median (Q1–Q3)	7.25 (6.52–8.09)	7.32 (6.74–7.96)	
LCn3			0.94 ^1^
Mean (SD)	7.10 (1.16)	7.11 (1.02)	
Median (Q1–Q3)	6.90 (6.25–7.75)	7.05 (6.50–7.66)	
EPA			0.03 ^2^
Mean (SD)	0.73 (0.23)	0.80 (0.25)	
Median (Q1–Q3)	0.65 (0.55–0.82)	0.77 (0.62–0.89)	
DHA			0.60 ^1^
Mean (SD)	3.92 (0.96)	3.83 (0.82)	
Median (Q1–Q3)	3.79 (3.25–4.61)	3.73 (3.34–4.48)	
Total n6			0.49 ^1^
Mean (SD)	26.44 (1.48)	26.61 (1.39)	
Median (Q1–Q3)	26.69 (25.43–27.40)	26.63 (25.69–27.50)	
n6/n3 ratio			0.99 ^1^
Mean (SD)	3.68 (0.71)	3.68 (0.65)	
Median (Q1–Q3)	3.69 (3.20–4.26)	3.61 (3.28–4.04)	
**Quality of life characteristics**			
**EPIC-26**			
Urinary incontinence			0.30 ^2^
Mean (SD)	92.61 (13.30)	93.76 (13.39)	
Median (Q1–Q3)	100.00 (91.75–100.00)	100.00 (100.00–100.00)	
Missing n	4 (6.15)	3 (4.61)	
Urinary irritation			0.17 ^1^
Mean (SD)	83.97 (15.66)	87.50 (12.75)	
Median (Q1–Q3)	87.50 (75.00–93.75)	87.50 (81.25–100.00)	
Missing n	3 (4.61)	4 (6.15)	
Sexual			0.26 ^1^
Mean (SD)	62.45 (27.93)	68.05 (27.75)	
Median (Q1–Q3)	58.33 (40.33–87.50)	77.08 (48.66–87.50)	
Missing n	2 (3.07)	3 (4.61)	
Hormonal			0.42 ^2^
Mean (SD)	89.07 (16.06)	89.19 (12.45)	
Median (Q1–Q3)	95.00 (80.00–100.00)	90.00 (85.00–100.00)	
Missing n	3 (4.61)	3 (4.61)	
Bowel			0.08 ^2^
Mean (SD)	90.71 (13.40)	93.88 (11.08)	
Median (Q1–Q3)	95.83 (87.50–100.00)	100 (91.66–100.00)	
Missing n	4 (6.15)	3 (4.61)	
**IPSS**			
Mean (SD)	8.70 (6.72)	8.22 (5.98)	0.59 ^1^
Median (Q1–Q3)	7.00 (3.00–13.00)	7.00 (4.00–11.00)	
Missing n	1 (1.53)	2 (3.07)	

**Notes:***p*-values were obtained using ^1^ student *t*-test, ^2^ Wilcoxon test, or ^3^ chi-2 test. * Red blood cells fatty acid profile is expressed as a percentage of total fatty acids. SD: standard deviation; Q1: lower quartile; Q3: upper quartile; BMI: body mass index; PSA: prostate specific antigen; NCCN: National Comprehensive Cancer Network, RBC: red blood cells; n3: omega-3 fatty acids; n6: omega-6 fatty acids; LCn3: long-chain omega-3 fatty acids; EPA: eicosapentaenoic acid; DHA: docosahexaenoic acid; EPIC-26: The Expanded Prostate Cancer Index Composite; IPSS: The International Prostate Symptom Score.

**Table 2:** Associations between quality-of-life functions and MAG-EPA intervention, intention-to-treat analysis.

Variables	Placebo			MAG-EPA			Difference between Group	
	Mean (SE)	MD (95% CI)	*p*-Value *	Mean (SE)	MD (95% CI)	*p*-Value *	MD (95% CI)	*p*-Value **
**EPIC-26**								
Urinary Incontinence								
Randomization	94.7 (2.0)	0.0		94.5 (1.9)	0.0			
3 Months Post-RP	47.9 (3.4)	−46.8 (−54.3; −39.4)	<0.0001	36.8 (3.3)	−57.6 (−65.1; −50.1)	<0.0001	−9.8 (−19.2; −0.4)	0.04
6 Months Post-RP	66.7 (3.6)	−28.0 (−36.0; −20.1)	<0.0001	58.6 (3.7)	−35.8 (−43.9; −27.8)	<0.0001	−6.6 (−16.6; 3.5)	0.20
9 Months Post-RP	69.3 (3.5)	−25.4 (−32.8; −18.1)	<0.0001	67.0 (3.5)	−27.4 (−35.0; −19.9)	<0.0001	−0.5 (−10.0; 9.0)	0.92
12 Months Post-RP	73.2 (3.5)	−21.5 (−28.7; −14.3)	<0.0001	66.5 (3.6)	−27.9 (−35.4; −20.5)	<0.0001	−3.7 (−13.4; 6.0)	0.45
Urinary irritation								
Randomization	85.6 (2.1)	0.0		83.1 (2.0)	0.0			
3 Months Post-RP	78.5 (2.1)	−7.1 (−11.9; −2.2)	0.005	79.1 (2.0)	−4.1 (−8.9; 0.8)	0.10	1.6 (−3.9; 7.1)	0.57
6 Months Post-RP	85.3 (1.8)	−0.3 (0.1; 8.8)	0.90	87.8 (1.8)	4.7 (0.4; 8.9)	0.03	2.6 (−1.8; 7.0)	0.24
9 Months Post-RP	86.9 (1.8)	1.4 (−2.3; 5.1)	0.47	87.3 (1.8)	4.1 (0.4; 7.8)	0.03	1.7 (−2.4; 5.8)	0.42
12 Months Post-RP	86.6 (1.7)	1.0 (−3.0; 5.1)	0.62	88.8 (1.7)	5.6 (1.5; 9.6)	0.007	3.5 (−0.8; 7.8)	0.11
Sexual function								
Randomization	65.7 (3.7)	0.0		62.8 (3.6)	0.0			
3 Months Post-RP	19.6 (2.8)	−46.1 (−53.4; −38.9)	<0.0001	17.7 (2.8)	−45.1 (−52.3; −37.8)	<0.0001	−1.8 (−9.0; 5.4)	0.62
6 Months Post-RP	26.3 (3.3)	−39.4 (−46.8; −31.9)	<0.0001	26.2 (3.3)	−36.6 (−44.1; −29.2)	<0.0001	−0.4 (−8.6; 7.9)	0.93
9 Months Post-RP	32.0 (3.5)	−33.7 (−40.8; −26.6)	<0.0001	30.5 (3.5)	−32.3 (−39.5; −25.2)	<0.0001	−1.8 (−10.4; 6.7)	0.67
12 Months Post-RP	36.0 (3.8)	−29.7 (−37.2; −22.3)	<0.0001	31.5 (3.8)	−31.3 (−38.8; −23.8)	<0.0001	−4.5 (−14.0; 5.0)	0.35
Bowel function								
Randomization	92.8 (1.7)	0.0		90.2 (1.6)	0.0			
3 Months Post-RP	91.3 (1.6)	−1.5 (−5.2; 2.2)	0.42	89.7 (1.7)	−0.5 (−4.2; 3.2)	0.77	−0.8 (−5.2; 3.7)	0.72
6 Months Post-RP	93.0 (1.4)	0.2 (−2.8; 3.2)	0.90	91.4 (1.3)	1.2 (−1.8; 4.3)	0.43	−1.0 (−4.3; 2.3)	0.55
9 Months Post-RP	93.0 (1.7)	0.2 (−3.4; 3.8)	0.93	90.8 (1.7)	0.6 (−3.2; 4.2)	0.76	−0.4 (−4.5; 3.7)	0.84
12 Months Post-RP	95.2 (1.3)	2.4 (−0.5; 5.2)	0.11	94.0 (1.2)	3.8 (0.9; 6.7)	0.009	−0.6 (−3.4; 2.2)	0.69
Hormonal function								
Randomization	85.9 (2.0)	0.0		88.3 (1.9)	0.0			
3 Months Post-RP	85.8 (1.9)	−0.1 (−3.8; 3.6)	0.96	88.2 (1.8)	−0.1 (−3.8; 3.7)	0.97	0.9 (−3.4; 5.3)	0.66
6 Months Post-RP	88.1 (1.6)	2.2 (−1.3; 5.8)	0.21	88.2 (1.5)	−0.1 (−3.6; 3.4)	0.96	−0.3 (−3.9; 3.4)	0.89
9 Months Post-RP	87.5 (1.6)	1.6 (−1.7; 4.9)	0.33	89.4 (1.5)	1.2 (−2.2; 4.5)	0.49	1.4 (−2.2; 5.0)	0.43
12 Months Post-RP	86.6 (1.7)	0.7 (−2.5; 4.0)	0.66	89.2 (1.7)	1.0 (−2.2; 4.2)	0.55	1.6 (−2.0; 5.3)	0.36
**IPSS**								
Voiding and storage Problem								
Randomization	9.0 (0.9)	0.0		8.8 (0.8)	0.0			
3 Months Post-RP	11.2 (0.9)	2.2 (0.1; 4.3)	0.04	10.3 (0.9)	1.5 (−0.6; 3.6)	0.17	−0.9 (−3.2; 1.4)	0.43
6 Months Post-RP	7.7 (0.7)	−1.2 (−3.0; 0.5)	0.17	7.8 (0.7)	−1.0 (−2.7; 0.8)	0.28	0.1 (−1.6; 1.8)	0.91
9 Months Post-RP	7.3 (0.7)	−1.7 (−3.3; 0.0)	0.05	6.5 (0.7)	−2.3 (−4.0; −0.6)	0.007	−0.9 (−2.7; 0.8)	0.30
12 Months Post-RP	6.9 (0.7)	−2.1 (−3.8; −0.4)	0.02	5.9 (0.6)	−2.9 (−4.6; −1.2)	0.0008	−1.1 (−2.8; 0.5)	0.17

Notes: MAG-EPA: monoacylglyceride-conjugated eicosapentaenoic acid; RP: radical prostatectomy. * *p*-value were obtained by mixed models adjusted for age. BMI and NCCN risk; ** *p*-value were obtained by mixed models adjusted for age, BMI, NCCN risk, and baseline score.

**Table 3:** Associations between quality-of-life functions and MAG-EPA intervention, per-protocol analysis.

Variables	Placebo			MAG-EPA			Difference between Group	
	Mean (SE)	MD (95% CI)	*p*-Value *	Mean (SE)	MD (95% CI)	*p*-Value *	MD (95% CI)	*p*-Value **
**EPIC-26**								
Urinary Incontinence								
Randomization	94.5 (2.0)	0.0		94.2 (1.9)	0.0			
3 Months Post-RP	47.3 (3.7)	−47.2 (−55.3; −39.2)	<0.0001	37.1 (3.8)	−57.2 (−65.3; −48.9)	<0.0001	−7.2 (−17.9; 3.4)	0.18
6 Months Post-RP	66.4 (4.1)	−28.1 (−36.9; −19.3)	<0.0001	58.3 (4.3)	−35.9 (−45.2; −26.7)	<0.0001	−4.7 (−16.5; 7.2)	0.44
9 Months Post-RP	70.8 (3.8)	−23.7 (−31.6; −15.7)	<0.0001	65.7 (4.1)	−28.5 (−37.1; −19.9)	<0.0001	−1.4 (−12.6; 9.7)	0.80
12 Months Post-RP	74.4 (3.9)	−19.1 (−28.0; −12.1)	<0.0001	65.8 (4.1)	−28.6 (−37.1; −20.2)	<0.0001	−4.4 (−15.9; 7.2)	0.45
Urinary irritation								
Randomization	85.2 (2.1)	0.0		82.5 (2.0)	0.0			
3 Months Post-RP	78.9 (2.3)	−6.3 (−11.4; −1.3)	0.01	77.0 (2.3)	−5.4 (−10.5; −0.3)	0.04	0.3 (−5.9; 6.4)	0.93
6 Months Post-RP	85.1 (2.0)	−0.1 (−4.5; 4.3)	0.95	85.8 (2.0)	3.3 (−1.2; 7.7)	0.15	2.1 (−2.9; 7.0)	0.41
9 Months Post-RP	87.5 (1.9)	2.3 (−1.3; 6.0)	0.21	85.3 (1.9)	2.9 (−0.8; 6.5)	0.12	0.7 (−3.8; 5.1)	0.77
12 Months Post-RP	84.4 (2.0)	−0.8 (−5.0; 3.5)	0.72	87.3 (2.0)	4.9 (0.6; 9.2)	0.03	5.5 (0.4; 10.6)	0.03
Sexual function								
Randomization	66.3 (3.6)	0.0		63.2 (3.5)	0.0			
3 Months Post-RP	19.0 (3.0)	−47.3 (−54.6; −40.0)	<0.0001	18.8 (3.0)	−44.4 (−51.7; −37.1)	<0.0001	−0.7 (−8.5; 7.1)	0.86
6 Months Post-RP	28.1 (3.6)	−38.2 (−45.8; −30.6)	<0.0001	27.4 (3.8)	−35.8 (−43.6; −28.0)	<0.0001	−1.2 (−10.6; 8.2)	0.80
9 Months Post-RP	33.0 (3.8)	−33.3 (−40.8; −25.8)	<0.0001	31.4 (4.0)	−31.8 (−39.6; −24.1)	<0.0001	−2.3 (−12.2; 7.6)	0.65
12 Months Post-RP	36.2 (4.3)	−30.1 (−38.1; −22.2)	<0.0001	32.4 (4.5)	−30.8 (−39.0; −22.6)	<0.0001	−3.8 (−15.1; 7.5)	0.50
Bowel function								
Randomization	93.1 (1.7)	0.0		90.3 (1.6)	0.0			
3 Months Post-RP	92.8 (1.8)	−0.3 (−4.3; 3.7)	0.86	88.5 (1.8)	−1.8 (−5.8; 2.2)	0.38	−3.2 (−8.2; 1.7)	0.20
6 Months Post-RP	93.6 (1.4)	0.5 (−2.6; 3.7)	0.75	91.5 (1.4)	1.2 (−1.9; 4.5)	0.45	−1.6 (−5.2; 2.0)	0.37
9 Months Post-RP	94.3 (1.7)	1.2 (−2.8; 5.2)	0.56	89.9 (1.8)	−0.4 (−4.5; 3.7)	0.85	−2.4 (−6.8; 2.0)	0.28
12 Months Post-RP	96.2 (1.3)	3.0 (−0.3; 6.4)	0.07	93.9 (1.3)	3.6 (0.3; 6.9)	0.03	−1.0 (−4.4; 2.4)	0.55
Hormonal function								
Randomization	86.3 (2.0)	0.0		87.9 (1.9)	0.0			
3 Months Post-RP	88.0 (1.9)	1.6 (−1.4; 4.6)	0.29	87.2 (1.9)	−0.7 (−3.8; 2.4)	0.64	−1.2 (−5.2; 2.9)	0.57
6 Months Post-RP	88.7 (1.5)	2.4 (−0.3; 5.1)	0.08	89.2 (1.6)	1.3 (−1.4; 3.9)	0.35	−0.2 (−3.2; 2.8)	0.90
9 Months Post-RP	87.8 (1.6)	1.4 (−1.8; 4.6)	0.38	88.6 (1.7)	0.7 (−2.5; 3.9)	0.66	0.5 (−3.3; 4.3)	0.80
12 Months Post-RP	88.3 (1.7)	1.9 (−1.3; 5.1)	0.24	90.4 (1.7)	2.5 (−0.7; 5.7)	0.12	1.7 (−2.2; 5.6)	0.38
**IPSS**								
Voiding and storage Problem								
Randomization	9.1 (0.9)	0.0		9.0 (0.9)	0.0			
3 Months Post-RP	11.1 (1.0)	1.9 (−0.3; 4.2)	0.08	11.1 (1.0)	2.2 (−0.1; 4.4)	0.06	−0.3 (−2.9; 2.4)	0.85
6 Months Post-RP	7.8 (0.7)	−1.3 (−3.0; 0.5)	0.15	8.4 (0.8)	−0.6 (−2.4; 1.1)	0.48	0.2 (−1.6; 2.0)	0.81
9 Months Post-RP	7.0 (0.7)	−2.2 (−3.8; −0.5)	0.009	7.2 (0.8)	−1.8 (−3.5; −0.2)	0.03	−0.1 (−1.9; 1.7)	0.91
12 Months Post-RP	7.0 (0.7)	−2.1 (−3.8; −0.5)	0.01	6.4 (0.7)	−2.6 (−4.2; −0.9)	0.002	−0.9 (−2.8; 0.9)	0.30

Notes: MAG-EPA: monoacylglyceride-conjugated eicosapentaenoic acid; RP: radical prostatectomy. * *p*-value were obtained using mixed models adjusted for age. BMI and NCCN risk; ** *p*-value were obtained by mixed models adjusted for age, BMI, NCCN risk, and baseline score.

**Table 4:** Reported adverse events for the entire study.

	Placebo (n = 65) * n (%)	MAG-EPA (n = 63) * n (%)
Diarrhea	1 (1.5)	3 (4.8)
Skin rash	3 (4.6)	1 (1.6)
Nausea	1 (1.5)	3 (4.8)
Heartburn	0	1 (1.6)
Digestive problems	1 (1.5)	1 (1.6)
Change of stool frequency	1 (1.5)	1 (1.6)
Headaches	0	1 (1.6)
Total of patients with events	7 (10.8)	10 (15.9)
Withdrawal because of adverse events	1 (1.5)	5 (7.9)

Notes: * Adverse events are reported for all patients who had radical prostatectomy (n = 128). MAG-EPA: monoacylglyceride-conjugated eicosapentaenoic acid.

**Table 5:** Adherence to study drug.

	Placebo (n = 65)	MAG-EPA (n = 63)
Adherence to study drug for the entire study (%) *	91.9	93.3
Adherence to study drug until withdrawal or end of the study **	91.4	92.9
Adherence to study drug for all patients who underwent RP ^§^	86.3	83.4
Adherence for patients who underwent RP and took at least 80% of study drug ^†^	93.4	94.8

Notes: * Adherence to the study drug was calculated for the entire study for patients who completed the 12 Months Post-RP visit and had available compliance data for the entire study (n = 55 in placebo; n = 58 in MAG-EPA). ** Adherence to the study drug was calculated for all patients who had available data for compliance to the study drug before their withdrawal or the end of the study, whichever came first (n = 57 in placebo; n = 62 in MAG-EPA). § Adherence to study drug was calculated for all patients who underwent radical prostatectomy and had available data for compliance to study drug (n = 63 in placebo; n = 62 in MAG-EPA). For patients who withdrew from the study, subsequent visits compliance was considered to be 0%. † Adherence to the study drug was calculated for all patients who underwent radical prostatectomy and took at least 80% of the dose during the entire study (mean of all visits) (n = 54 in placebo; n = 52 in MAG-EPA). MAG-EPA: monoacylglyceride-conjugated eicosapentaenoic acid; RP: Radical prostatectomy.

## Data Availability

The data set generated and codes used during the current study are available from the corresponding author on reasonable request.
